# The efficacy and safety of uniportal video-assisted thoracic surgery on the treatment for stage II-III tuberculous empyema: a single-arm clinical retrospective study from 2016 to 2021 in a thoracic surgery center in China

**DOI:** 10.1186/s12890-022-02182-w

**Published:** 2022-11-03

**Authors:** Wenwen Sun, Guidong Yin, Haomin Cai, Yiming Zhou, Jin Gu, Shanhao Chen, Lin Fan

**Affiliations:** 1 Shanghai Clinic and Research Center of Tuberculosis, Department of Tuberculosis, Shanghai Key Laboratory of Tuberculosis, Shanghai Pulmonary Hospital, Tongji University School of Medicine, 200433 Shanghai, China; 2 Department of Thoracic Surgery, Changchun Infectious Disease Hospital,, Changchun, China; 3 Department of Thoracic Surgery, Shanghai Pulmonary Hospital, Tongji University School of Medicine, Shanghai, China

**Keywords:** Tuberculous empyema, Uniportal video-assisted thoracic surgery, Adverse reactions, Treatment outcome

## Abstract

**Background:**

Surgery is an important adjuvant treatment for tuberculous empyema(TE). We thus conducted a single arm-clinical retrospective study of stage II-III TE patients who underwent uniportal video-assisted thoracic surgery (Uni-VATS) over a 5-year period to evaluate the efficacy and safety of surgery on TE, so as to provide the evidence for the optimal clinical strategies.

**Methods:**

Patients diagnosed as TE with withdrawal of anti-tuberculosis-VATS were retrospectively enrolled from January 2016 to December 2021. All patients were followed up untill 12 months after withdrawal of anti-tuberculosis treatment (ATT). Clinical characteristics and surgical details were observed and analyzed to evaluate the efficacy and safety of the minimally invasive surgery.

**Results:**

Totally 439 cases met included criteria were enrolled, no deaths were reported. The mean operative time was 2.6 (1.9, 4.3) hours and the mean intraoperative blood loss was 356 (240, 940) ml. Blood transfusion was performed in 20.50% (90/439) of patients and additional pneumonectomy was occurred in 9.89%(37/439)of patients .The mean postoperative drainage time was 12 (7, 49) days and the mean hospital stay was 6 (4,12) days. All stage II TE achieved complete lung re-expansion after surgery while 84.22%(315/374) of stage III achieved complete lung re-expansion, *p* 0.00. 15.78% (59/374) of stage III TE achieved incomplete re-expansion, 4 of which underwent a second decortication by Uni-VATS. Recurrences rate was 2.96% (13/439), including 11 cases of early recurrence and 2 cases of late recurrence at TE stage III, 5 of which underwent a second decortication by Uni-VATS.

**Conclusion:**

Uni-VATS is highly effective safe and minimally invasive for patients with TE, which could be recommended as the mainstream operation in areas with high TB burden.

## Introduction

Tuberculosis (TB) is one of the most important causes of pleural infection worldwide, especially in TB endemic areas [1]. Tuberculous empyema(TE) is a chronic active pleural *mycobacterium tuberculosis*(MTB)infection with neutrophil infiltration and purulent effusion, culminating in extensive pleural thickening and calcification, which could have a lasting influence on lung function [2]. Treatment on TE has still been challenging due to its clinical complexity, which often requires multidisciplinary collaboration, including thoracic surgery.

During the fibrin purulent stage (stage II), fibrin deposition may lead to thoracic dissection and fluid separation, further limiting lung expansion [3]. Therefore, a simple closed thoracic drainage may not be sufficient, making local infection difficult to be controlled. Without aggressive intervention, empyema could progress to the tissue package stage (stage III), the stage is characterized by significant pleural thickening resulting from collagen fiber deposition, which further limits the movement of the lungs, chest wall and diaphragm [3]. On the other hand, the inadequate permeability of anti-tuberculous drugs into the thick fibrous calcified wall of empyema may further result in poor treatment efficacy [4]. The goals of surgical interventions include controlling the source of infection through complete pleural debridement and lung re-expansion. Current guidelines from the American Association of Thoracic Surgeons [5] and the British Society of Thoracic Surgeons [6] recommended that video-assisted thoracic surgery (VATS) or thoracotomy should be the first-line treatment for empyema if chest tube drainage fails. Traditionally, open thoracotomy (removal of thickened viscera and parietal pleura) was once the choice of intervention, especially for cases in tissue stage (stage III) TE [7]. Despite the high success rate of surgical interventions, mortality from thoracotomy surgery is also concerned, especially in the elderly and patients with complications. Minimally invasive surgeries are developing in recent years, which had been proven to be safe and effective in the adjuvant treatment for TE [8]. VATS provides a minimally invasive approach with one to three lateral openings, requiring fewer hospital stays and complications than thoracotomy [5]. Recent data had demonstrated the positive effects of VATS decortication on TE [9]. If necessary, the surgeon can immediately switch to open thoracotomy to achieve the same goal. Uniportal-VATS(Uni-VATS)has been considered to be the least invasive thoracic surgical procedure, prompting its worldwide adoption [10]. The application of the Uni-VATS for the treatment of pleural infection has not been well identified, but it could be useful to minimize surgical trauma. Recent literature suggests that early and aggressive minimally invasive approaches to stage II empyema can rapidly relieve infection and ensure lower morbidity, shorter hospital stay and lower costs. However, VATS in advanced tissue stage(stage III)empyema still remains controversial [11]. In the present study, we retrospectively enrolled the patients with TE at stage II-III receiving Uni-VATS during the recent five years, and evaluated the efficacy and safety of the surgery, thus try to provide guidance for optimal surgical treatment for TE patients in high TB burden areas.

## Materials and methods

### Ethics statement

The study was conducted at a national TB clinical research center with nearly 300 beds that received referrals for patients mainly from east China. Established in 1953, the thoracic surgery department of the hospital is a national key clinical specialty with 279 beds and over 20,000 operations annually. Currently, it is the world’s largest general thoracic surgery center with the largest amount of minimally invasive surgery. The study was approved by the Ethics Committee of shanghai pulmonary hospital (Approval No L20-357).

### Study design

This is a retrospective study using a prospective database of consecutively enrolled patients who underwent Uni-VATS for TE at stage II-III from January 2016 to December 2020. We obtained and recorded the following information: general information, clinical features including imaging, microbiology, pleural tissue histopathology, biochemistry, serology, surgical records, anti-tuberculosis treatment (ATT), etc.

The natural evolution of pleural effusion was divided into three stages: the first stage was stage I with pathological change of exudation, then was stage II with characteristics of fibrin purulence, the last was stage III (tissue stage). In the fibrin purulent stage(stage II), the pleural fluid appears purulent. This stage is characterized by positive microbiology in pleural effusion result, fibrin deposition and the color of the effusion may progress from a clear yellow to a suppurative opacity. Biochemical analysis of pleural fluid showed glucose < 40 mg/dL and LDH value > 1000 IU/L [3, 5, 12]. The fibrin purulent stage is followed by the tissue stage (stage III), where solid pleural dissection replaces the soft fibrin covering the visceral pleura which limits the lungs expansion. The most relevant clinical feature of stage III TE is the fibrous pleural skin covering the visceral pleura and parietal pleura [3, 5, 12].

The patients met all the following inclusion criteria were included in the study: 1)Patients were diagnosed as TE at stage II or III having surgical indications. 2) Empyema drainage clearance of pleural cavity decortication when necessary were performed in all patients through Uni-VATS. 3) All patients were followed up regularly for more than 1 year after withdrawal of ATT, and complete clinical data were retained.

Exclusion criteria: (1) Patients with a postoperative diagnosis of non-tuberculous empyema. (2) Patients unable to complete ATT due to severe adverse reactions. (3) Patients with incomplete clinical follow-up.

The surgical indications were determined by experienced pulmonary specialists, infectious specialists, and surgeons: TE at Stage II and III, characterized by pleural thickening leading to limited lung dilation, empyema with or without bronchopleural fistula (BPF), progression or recurrence after effective ATT [13].

Finally, 455 cases with complete clinical data undergoing Uni-VATS were firstly screened, among which 13 cases were excluded due to lost of follow-up and 3 cases were changed to thoracotomy during surgery. Finally, 439 cases were included. All empyema operations in our hospital are performed by the same surgeon.

### Uni-VATS

**Fig. 1:**
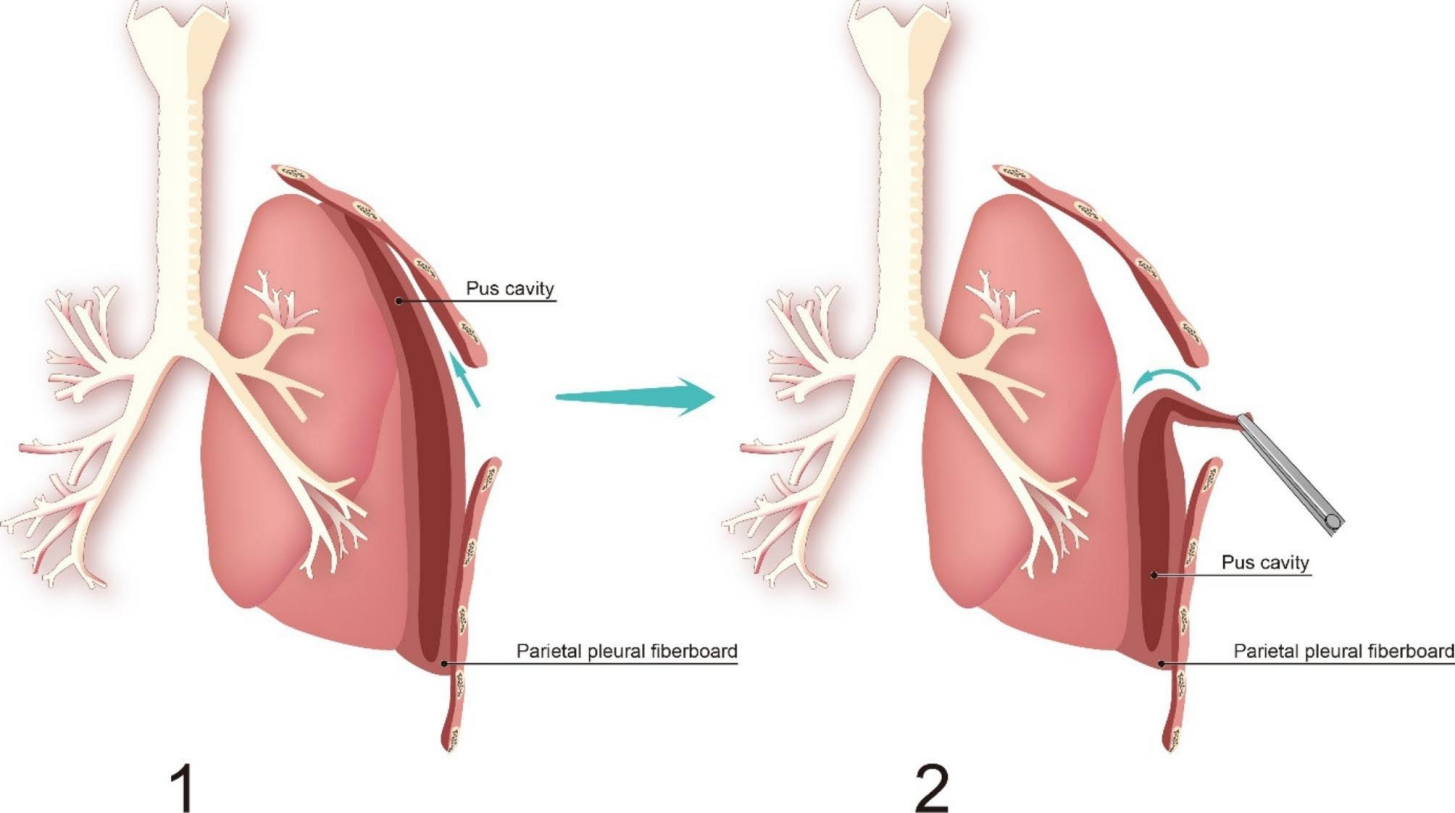
Thoracoscopic pleural exfoliation

The patient underwent standard double-lumen endotracheal intubation under general anesthesia. The location of the incision was selected according to the pre-operative CT. After blunt dissection, gauze was used to fill the gap between the fibrous pleural skin and the chest wall, and pressure was applied to stop bleeding. The wound protector was used to further enlarge the incision. Part of the parietal fiber pleura under the incision was resected. Various microbiological tests were performed after the pus was sucked out of the chest cavity. Then a careful incision was made to find the pleura between the fibrous pleura epidermis and the viscera, and the two layers were bluntly separated. After visceral fibrous pleural excision, adhesions between the lungs and the anterior and posterior mediastinum were removed. Try to isolate the lungs. After dissection of the surrounding visceral pleura, blunt dissection was performed on the site below the incision to separate the mural fiber pleura skin. Then blunt separation continues to the junction to the fibrous pleural epidermis and lung surface. Finally, the fibrous pleural epidermis was separated by blunt dissection. After all fibrous pleura were removed, the thorax was repeatedly irrigated with normal saline for careful hemostasis (Fig. 1**).** At the end of the procedure, major air leaks were repaired with sutures or surgical stents and extensive drainage was performed with 2 chest tubes.

### Preoperative/postoperative treatment and follow-up

The patients enrolled all received ATT before surgery. The average preoperative duration of ATT for stage II TE was 6 (4,12) weeks, and that for stage III TE was 19 (12, 30) weeks. All patients underwent etiological testing, inculding drug sensitivity test (DST) based on culture and Xpert MTB/RIF (Xpert), on respiratory specimens/pleural effusion/ pleural tissue. The standard ATT regimen (isoniazid, rifampicin, ethambutol, pyrazinamide) was used in patients without evidence of drug-resistance. For patients with evidence of drug-resistance based on DST, the regimens were made according to the WHO guideline [14]. After operation, patients continued the ATT and might adjust the regimens if drug-resistance results showed on postoperative specimens. The volume and color of chest tube drainage were monitored daily and air leakage was measured by a digital drainage device. After no air leakage was observed and the volume of drainage was less than 50 ml/day, chest CT was reviewed and the drainage tube could be removed. All patients were followed up for 1 year after drug withdrawal.

The recent efficacy was determined by postoperative lung re-expansion radiographically. Complete lung re-expansion is characterized by complete lung dilation with no residual collection or pleural thickening on at least two postoperative chest CT images. Incomplete lung re-expansion was defined as lung collapse on CT image with or without cardiac or costophrenic angle passivation/effusion. Failure of lung re-expansion is characterized by no change in postoperative CT compared with preoperative CT [15].

### Statistical analysis

SPSS 17.0 (SPSS Inc, Chicago, IL) was used for statistical analysis. Continuous variables were expressed as mean ± standard deviation or median. Categorical variables were expressed in rates and percentages. The student’s T-test was used to compare the continuous variables of normal distribution groups. χ2 test or Fisher’s exact test were used to compare the nominal classification data between groups. Mann-Whitney U test was used to compare non-normally distributed continuous variables. A *p*-value less than 0.05 was considered statistically significant.

## Results

### General information of patients enrolled

A total of 439 cases were enrolled, according to the stage of empyema (stage II and III), the patients enrolled were divided into two groups, 65 cases at stage II and 374 cases at stage III. No mortality was reported in the study population. Of all TE patients who underwent surgery, 78.8% (346/439) were male, accounting for 69.2% (45/65) in stage II and 80.5% (305/374) in stage III. However, there was no significant difference in gender distribution between the two groups (*p* 0.06). The average age in the stage III group was significantly older than that in the stage II group (*p* 0.01). Complications with diabetes were rare, especially for stage II. There was no significant difference in the incidence of the right side and left side between two groups (*p* 0.84), but the TE patients with bilateral empyema were all at stage III. Patients with symptoms lasting longer than 6 months mainly developed stage III TE, *p* 0.01. Most stage II patients were newly diagnosed (76.92%), while most stage III patients were retreated (86.97%), *p* 0.00. The mean duration of preoperative ATT was 6（4,12） weeks in stage II patients and 19 (12, 30) weeks in stage III patients, *p* 0.01.

Drug-resistant TE was rare, with a total of 11 cases, and mainly occurs in stage III (9/11, 81.82%). In the present study, the TE cases with drug-resistance were confirmed by culture or molecular drug-susceptibility test(DST) on pleural or respiratory specimens resistant to any anti-TB drug, including isoniazid- mono -resistance TE (n = 2), multidrug-resistant(MDR)/RR-TB-TE (n = 7), pre-extensively drug-resistance TE (pre-XDR, n = 1) and extensively drug-resistance TE (XDR, n = 1). The definitions of XDR-TB and Pre-XDR-TB were according to the guideline of WHO 2016 [14]. Two patients with stage II TE were both isoniazid-monoresistant TE.

The decline of lung function caused by empyema may mainly occur in stage III cases, *p* 0.02. And all patients with BPF were at stage III TE. The clinical details were shown in Table 1.

**Table 1:** General information of patients enrolled

Clinical details	Stage II empyema n = 65	Stage III empyema n = 374	***P***
Sex (n,%)			
Male	45 (69.23)	301(80.48)	0.06
Femaile	20(30.77)	73 (19.52)	
Age(years, median )	29 (20, 45)	41 (25, 68)	0.01*
Diabetes (n,%)	2(3.08)	30 ( 8.02)	0.25
Duration of clinical symptoms			
>6 months (n,%)	5(7.69)	82 (21.92)	0.01*
Location of disease			
Left	28(43.08)	166 (44.39)	0.84
Right	37(56.92)	200 (53.48)	
Both sides	0(0.00)	8(2.13)	
History of *ATT			
Newly-diagnosed	50(76.92)	45(12.03)	0.00**
Retreated	15(23.08)	329 (87.97)	
Duration of ATT* before surgery (weeks, Median)	6 (4 ,12)	19 (12, 30)	0.01**
Bronchopleural fistula	0 (0.00)	45 ( 12.03%)	0.00**
Drug-resistant TB (n,%)	2 (3.08)	9 (2.41)	0.91
Lung function(Percentage of predicted values, %)			
FEV1*	60.7% ± 10.7%	49.1% ± 18.4%	0.02**
FVC*	70.5% ± 13.6%	62.5% ± 15.1%	

*ATT: anti tuberculosis treatment; *FEV1:forced expiratory volume in 1 s; *FVC: functional vital capacity: **The difference was statistically significant.

### Perioperative details

The general details of surgery were discussed in Table 2. No patients in the study population required conversion to thoracotomy, and no deaths were reported. The mean operative time was 2.6 (1.9, 4.3) hours, and the mean intraoperative blood loss was 356 (240, 940) ml. Blood transfusion was performed in 8.66% (38/439) of patients and additional pneumonectomy was occurred in 9.89%(37/439)of patients. The mean postoperative drainage time was 12 (7,49) days, and the mean hospital stay was 6 (4,12) days.

**Table 2:** Perioperative Details of surgery

Perioperative Details	Patients n = 439	Stage II n = 65	Stage III n = 374	***P***
Operative time(hours)	2.6(1.9, 4.3)	2.2(1.8, 3.1)	3.2(2.0, 5.1)	0.02*
Intraoperative blood loss(ml)	356 (240, 940)	300 (200, 800)	490 (370, 1500)	0.01*
Blood transfusion (n,%)	38 (8.66 )	2 (3.08)	36 (9.63)	0.03*
Hospital stay(days)	6 (4,12)	4(4,6)	7 (5,12)	0.03*
Additional pneumonectomy (n,%)	37 (9.89)	0	37(9.89)	0.00*
wedge resection	28	0 (0.00)	28 (7.49)	0.00*
lobectomy	9	0 (0.00)	9 (2.41)	
Duration of chest tube drainage(days)	12 ( 7,49)	10(7,12)	16 (14, 49)	0.01*
Lung re-expansion (n,%)				
Complete re-expansion	380 ( 86.56)	65(100.00)	315 (84.22)	0.00*
Incomplete re-expansion	59(13.44)	0(0.00)	59 (15.78)	
Persistent air leak (n,%)				
5–14 days	379 ( 86.33)	60 ( 92.31)	319 (85.29)	1.75
> 14 days	55 ( 12.53)	0 (0.00)	55 ( 14.71)	0.00*
Improvement in lung function(mean)				
FEV1	10.3 ± 3.9	8.2 ± 3.2.	13.4 ± 5.3	0.03*
FVC	13.6 ± 4.2	9.5 ± 2.9	17.7 ± 5.5	0.02*
Recurrence (n,%)				
Early recurrence	11(2.51)	0 (0.00)	11(2.94)	0.33
Late recurrence	2 ( 0.46)	0 (0.00)	2(0.53)	0.68

* The difference was statistically significant

Grouping statistics of stage II and III showed that: the mean operative time of stage II was 2.2 (1.8, 3.1) hours, significantly shorter than that of Stage III 3.2 (2.0, 5.1) hours, *p* 0.02. The mean intraoperative blood loss of stage II was 300 (200, 800) ml, significantly less than that of stage III 480 (370, 1500) ml, *p* 0.01. Intraoperative transfusion was required in 9.63%(36 /374)of stage III TE, significantly more than that in stage II ( *p* 0.04 ), accounting for 94.74%(36/38)of the total blood transfusions. In terms of hospital stay, stage III TE was significantly longer than that of stage II, *p* 0.03. The postoperative chest tube drainage time of stage II TE was 17 (14,21) days, significantly shorter than that of stage III 30 (21, 69) days, *p* 0.01. All additional pneumonectomy occurred in stage III TE, with wedge resection in 28 cases and lobectomy in 9 cases.

### Surgical efficacy and adverse events

The surgical efficacy and adverse reactions were also shown in Table 2. 86.56%(380/439)of the patients achieved complete lung re-expansion. 98.86% (434/439) of enrolled patients experienced air leakage for more than 5 days, of which 87.32% (379/434) experienced air leakage for (5-14) days.

Grouping statistics of stage II and III showed that: all stage II TE achieved complete lung re-expansion after surgery, while 84.22%(315/374)of stage III achieved complete lung re-expansion, *p* 0.00. 15.78% (59/374) cases of stage III TE achieved incomplete re-expansion, 4 of which underwent a second decortication by Uni-VATS for recovery. There was no significant difference between stage II and stage III in terms of air leakage over (5-14) days (*p* 1.75), but all air leakage over 14 days occurred in stage III empyema(*p* 0.00). The lung function of stage III TE was significantly improved compared with that of stage II after operation, *p* 0.03. All recurrences were occurred in stage III with 11 cases of early recurrence and 2 cases of late recurrence.

### Recurrence and patients undergoing a second operation

Recurrence occurred in 13 stage III TE patients, 11 of whom had early recurrence and 2 of whom had late recurrence. All of the patients with early recurrence had increased pleural effusion after extubation (confirmed by CT scan), accompanied by fever or cough. Four patients underwent a second thoracoscopic operation, and seven patients underwent CT-guided needle-chest tube placement and electronic drainage flask drainage. All patients underwent intensive ATT according to the results of DST, of which 2 patients had drug-resistant TE discovered after the first operation. Two patients had late recurrence, one with increased pleural effusion detected by CT six months after surgery, and the other with pleural tuberculoma eight months after surgery. The first case improved after thoracic closed drainage and intensive ATT. The patient with pleural tuberculoma underwent a second thoracoscopic exfoliation.

The other 4 patients who underwent the second thoracoscopic surgery were due to persistent air leakage lasting more than 14 days, and 2 of them lasted more than 30 days, 1 of them was drug-resistant TE that was not detected before the first operation.

## Discussion

Management of TE is challenging due to extensive pleural thickening and/or chest constriction. A multidisciplinary approach is often required, including effective ATT, adequate chest drainage nutritional support surgical pleural dissection to remove residual pus reduction of bacterial load and removal of thickened pleura to achieve lung re-expansion [12]. Conventional closed thoracic drainage may be ineffective because of localized fibrin suppurative deposits in stage II/III TE. The presence of multiple fibrous septa in the thorax leads to poor fluid drainage, further preventing the control of local inflammation and the complete elimination of systemic infection. Therefore, surgery is often required to assist in the complete elimination of the infection based on effective ATT [16]. Thoracic surgery in chronically infected conditions is technically more challenging because of the potential for severe adhesions and fibrosis. Complete exfoliation, that is, removal of all fibrotic tissue that constricts the lung, is critical for successful surgical treatment of stage III TE. Any residual cortex on the lung surface prevents it from fully reexpanding, leaving a cavity in the chest and leading to repeated pleural effusion, which is a high risk of re-infection. Therefore, thoracotomy with complete removal of thickening and parietal pleura has traditionally been the preferred surgical approach, especially for patients with tissue stage III TE [7, 17]. However, existing studies have shown that thoracotomy may cause more surgery-related adverse reactions and even mortality than minimally invasive surgery, and bring greater trauma to patients[5, 6]. With the rapid development of minimally invasive surgery, thoracoscopy has been shown to be useful as an adjunctive treatment for empyema [8], while Uni-VATS is considered the least invasive thoracoscopy [10]. Uni-VATS could reduce postoperative pain and facilitate postoperative recovery by confining surgical trauma to one intercostal space [18]. Therefore, we reviewed 5 years of clinical data from a large pulmonary and TB specialist hospital and summarized all cases of TE undergoing Uni-VATS to provide evidence for the selection of TE surgery in areas with high TB burdens.

In our study, patients who met the inclusion criteria underwent Uni-VATS pleural decortication and drainage, 85.19% (374/439) of whom were at stage III TE and some were accompanied with complex pleural conditions (such as BPF), but only 3 of them were converted to thoracotomy, and the remaining procedures were successfully completed by thoracoscopy. None of the patients died or failed treatment by the end of the study. Although a few patients (2.05%, 9/439) required a second thoracoscopic surgery, all patients were cured with no recurrence in 1-year follow-up after drug withdrawal. Our results confirm that Uni-VATS may be the mainstream surgical approach for TE in most cases, with satisfactory outcomes in both stage II and stage III. From the perspective of clinical characteristics, most of the TE patients requiring surgery were male, especially with stage III empyema accounted for 80.48%. This is similar to the previous study [19]. Our results also showed that compared with stage II, stage III TE had a significantly longer duration required longer preoperative ATT, and was more common in retreated population. The decline of lung function and BPF were mainly occurred in stage III TE. These clinical features are all similar to existing research results(8, 19), reflecting the clinical complexity of stage III TE, which determines the necessity and difficulty of operation.

The recent secondary surgery was performed in patients with incomplete lung re-expansion due to in-exhaustive fiberboard dissection or uncontrolled chest infection that failed to respond to ATT. In the present study, 9 cases of stages III TE patients underwent a second Uni-VATS procedure, of which 3 (33.3%) were undiagnosed preoperatively drug-resistant TE (27.3% of all drug-resistant TE). All patients were cured through the second procedure. Our result showed that Uni-VATS could still be used as a remedy for the failure of the first operation. Although in the present study, the operation time, intraoperative blood loss, hospital stay and postoperative drainage time of stage III TE were significantly longer than those of stage II TE. Additional pneumonectomy and recent secondary surgery were performed in some of the stage III TE patients. However, the lung function of stage III TE patients was significantly improved after surgery compared with stage II TE patients. This could be associated with a significant decrease in the preoperative lung function of stage III TE compared with stage II. The result suggested that stage III empyema may benefit more through surgery. Our results showed that all surgical adverse reactions and complications occurred in stage III TE, but were completely controllable. We therefore draw a preliminary inference that Uni-VATS is technically feasible for the treatment of complex TE.

Drug-resistant TB is now a growing burden around the world, including in China [20]. Although pleura TB is a common extra-pulmonary manifestation of drug-resistant TB [21], invasive surgical intervention may still be cautious. The main reason is that most patients diagnosed with Drug-resistant TE preoperatively may not have indications for minimally invasive surgery. For patients with extensive lesions, complex pleural conditions or poor response to ATT, minimally invasive surgery may not be as the the first choice. However, with the development of diagnosis and treatment of drug -resistant TB in recent years, we considered that it was possible to expand the indication of surgery for these patients [22]. We are also very interested in surgical treatment of drug-resistant TE and undergoing prospective studies to further improve the prognosis of this population.

Previous studies had shown that Uni-VATS decortication is feasible and safe, and had similar efficacy in infection control and recovery of lung function, with the advantage of lower adverse reactions and a better overall pleural display compared to thoracotomy [23, 24]. A recent consensus statement by European Association for Cardio-Thoracic Surgery (EACTS) concluded that VATS had an important role in the management of pleural empyema determined by its safety and effectiveness both in stages II and III, but thoracotomy still plays a fundamental role, especially for decortication [25]. However, most of the existing studies for diseases were mainly focused on chronic empyema, while the present study was focused on TE and yielded a similar result which represent the novelty of the study.

There were some limitations in our study. First of all, we enrolled patients in a hospital specializing in TB and pulmonary diseases with perfect ability of internal ATT and thoracic surgery. Thoracic surgical experience is considered to be a major factor in the outcome of VATS surgery [25]. Adequate ATT is the basis of successful surgery, which can be also achieved at the hospital. Therefore, our results may not be representative of widespread levels in areas with high TB prevalence but could serve as an achievable direction. Secondary, our study was a single-arm, retrospective study without a control group for thoracotomy, so it may be impossible to intuitively compare the advantages and disadvantages of the two surgical methods.

In conclusion, the main finding of the study was that Uni-VATS is minimally invasive, safe and highly effective for TE. For stage II TE, Uni-VATS has little trauma and quick recovery. For complex stage III TE, Uni-VATS could also achieve satisfactory outcome under safe and minimally invasive conditions in most cases. However, the important prerequisites for successful operation may be extensive experience in thoracoscopic surgery and effective ATT.

## Data Availability

All data regarding the included participants during the study are available from the corresponding author by email request.
